# Representation of prefrontal axonal efferents in the thalamic nucleus reuniens in a rodent model of fetal alcohol exposure during third trimester

**DOI:** 10.3389/fnbeh.2022.993601

**Published:** 2022-09-08

**Authors:** Eva A. Smith, Zachary H. Gursky, Anna Y. Klintsova

**Affiliations:** ^1^Department of Psychological and Brain Sciences, University of Delaware, Newark, DE, United States; ^2^Interdisciplinary Neuroscience Graduate Program, University of Delaware, Newark, DE, United States

**Keywords:** fetal alcohol, viral tracing, thalamus, nucleus reuniens, prefrontal cortex, unbiased stereology, microscopy

## Abstract

Alcohol exposure (AE) during the prenatal period could result in fetal alcohol spectrum disorders (FASDs), one of many deficits of which is impaired executive functioning (EF). EF relies on the coordination of activity between the medial prefrontal cortex (mPFC) and hippocampus (HPC) by the thalamic nucleus reuniens (Re), a structure that has been shown to be damaged following high-dose AE in a rodent model of third trimester exposure. Notably, mPFC neurons do not project directly to HPC, but rather communicate with it *via* a disynaptic pathway where the first cortical axons synapse on neurons in Re, which in turn send axons to make contacts with hippocampal cells. This experiment investigated the effect of binge AE (5.25 g/kg/day, two doses 2 h apart) during postnatal days 4–9 on the length of medial prefrontal axonal projections within Re in Long Evans rat. AE reduced the cumulative length of mPFC-originating axon terminals in Re in female rats, with male rats exhibiting shorter cumulative lengths when compared to female procedural control animals. Additionally, Re volume was decreased in AE animals, a finding that reproduced previously reported data. This experiment helps us better understand how early life AE affects prefrontal-thalamic-hippocampal connectivity that could underlie subsequent EF deficits.

## Introduction

Fetal alcohol spectrum disorders (FASDs), prevalent while completely preventable developmental disorders, can result from alcohol exposure (AE) during the prenatal period and affects between 1.1 and 5% of all children born in the United States (May et al., 2018). Individuals diagnosed with FASDs experience severe reductions of quality of life and long-lasting disabilities of cognition, motor control, emotionality, and social behavior (Streissguth et al., 1991). While the outcome of prenatal alcohol exposure has long been known for decades, public health efforts have still not reduced the frequency of alcohol use during pregnancy (Roozen et al., 2016).

**FIGURE 1:**
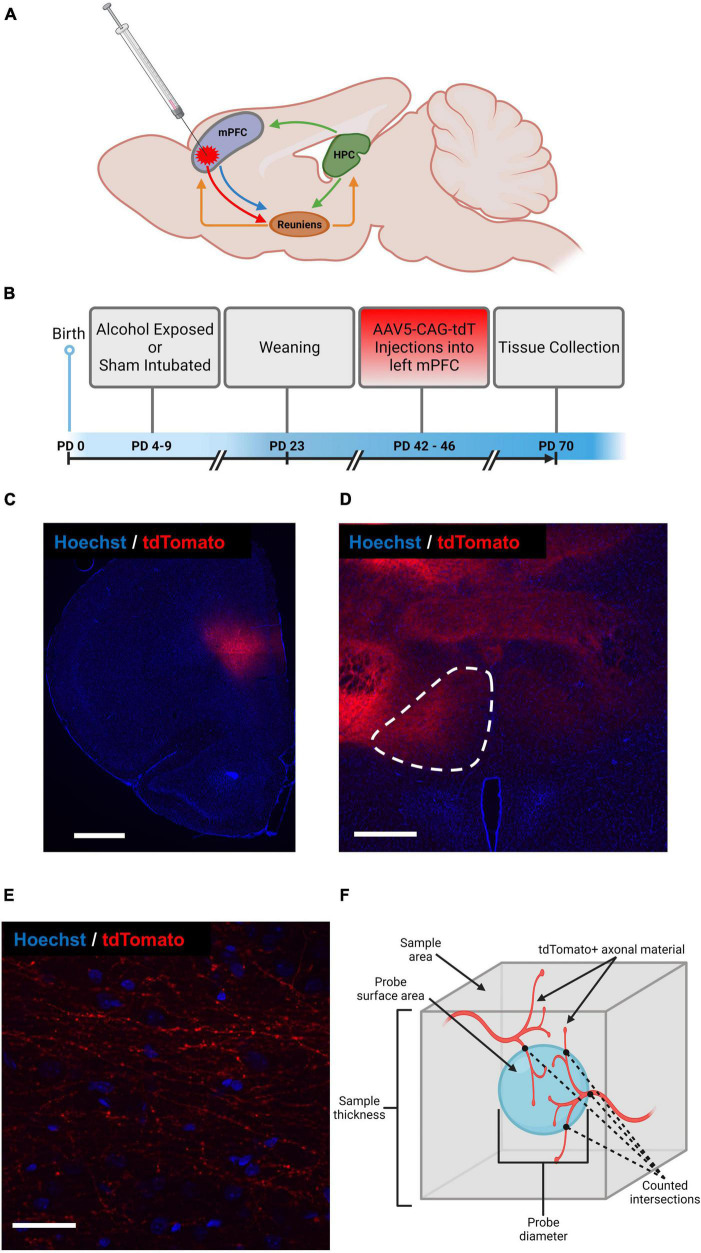
Schematic representation of mPFC-Re-HPC circuitry and indication of AAV-CAG-tdTomato injection site **(A)**, experimental timeline **(B)**, representative section containing AAV-CAG-tdTomato injection site into left mPFC (scale bar = 1,000 μm) **(C)**, representative section containing region of interest (i.e., left Re, outlined) (scale bar = 500 μm) **(D)**, magnified section of Re containing AAV-expressing axon terminals (scale bar = 40 μm) **(E)**, schematic representation of Spaceballs probe used to estimate cumulative length of AAV-expressing axon terminals in Re **(F)**. Figure created with BioRender.com.

While FASDs result in a variety of cognitive and behavioral deficits, one common impairment among alcohol-exposed individuals is that to executive functioning (EF) (Rasmussen, 2005; Rasmussen and Bisanz, 2009; Fuglestad et al., 2015). The underlying neuroanatomical substrates of EF in rodents include the medial prefrontal cortex (mPFC) and the hippocampus (HPC). It is important to note that, while the HPC makes direct connections to the mPFC, mPFC neurons do not project directly to HPC. Rather, projecting neurons in layers V/VI of mPFC communicate with HPC largely through a disynaptic pathway *via* neurons in the thalamic nucleus reuniens (Re) which, in turn, make contacts with HPC cells in the subiculum and CA1 regions (primarily ventral CA1) (McKenna and Vertes, 2004; Hoover and Vertes, 2012; Varela et al., 2014) (Figure 1A). Neurons in Re coordinate activity between mPFC and HPC and is a critical mediator of the mPFC-HPC pathway. Recent work has shown that Re is necessary for typical mPFC-HPC functional synchrony as well as successful spatial working memory in rats (Layfield et al., 2015; Hallock et al., 2016). Interestingly, children diagnosed with FASDs often display reduced thalamus size (Nardelli et al., 2011). These findings support the ventral midline thalamus as an integral part of EF-related circuitry.

Despite this evidence, mPFC-Re-HPC circuitry is understudied in regard to early life alcohol exposure. In fact, only four papers to date have addressed the effects of AE on this specific circuitry (Gursky et al., 2019, 2020b; Gursky and Klintsova, 2021, 2022). Indeed, work from our lab has shown that Re, but not neighboring thalamic nuclei such as the rhomboid nucleus, is specifically damaged in a rodent model of third-trimester binge drinking targeting the brain growth spurt (BGS). Both high- and moderate-dose AE results in reduced Re volume and reduced Re total neuron number in adulthood (Gursky et al., 2019; Gursky and Klintsova, 2022). Additionally, a single alcohol exposure on PD7 leads to a significant increase of apoptotic cell death in Re 12 h after exposure, displaying a high susceptibility of this structure to immediate alcohol exposure (Gursky et al., 2020b). More recently, our lab examined this effect of AE-induced neuron loss in relation to mPFC-Re-HPC circuitry by investigating the number of projection neurons in Re that synapse directly on mPFC or HPC by applying retrograde viral tracers into the mPFC and HPC. While AE reduced the total number of Re neurons, the proportion of remaining neurons that innervate both mPFC and HPC was larger in AE rats than in normal or procedural controls. However, AE had no significant effect on the proportion of remaining Re neurons that innervate only one of the two structures (Gursky and Klintsova, 2021).

In addition to significant impacts on Re and its efferent projections, AE has been shown to heavily impact the morphology of the mPFC. Indeed, AE has been reported to reduce both spine density and dendritic complexity in the mPFC (Whitcher and Klintsova, 2008; Hamilton et al., 2010; Lawrence et al., 2012). Furthermore, differential effects have been shown in a layer-specific manner, with layers II/III neurons exhibiting increased dendritic trees overall, while layer VI neurons exhibited extended long apical dendrites but decreased short apical dendrites (Louth et al., 2018). While most research to date has focused on the effects of AE on mPFC dendrites and not axons, this work supports different outcomes to mPFC neuron morphology in a layer-dependent manner after prenatal AE.

The purpose of this study was to examine the impact of AE on mPFC neuronal projections to Re, an integral part of mPFC-Re-HPC circuity, by labeling them with an anterograde viral tracer injected into the deep layers of the mPFC. Because of the extensive work demonstrating reduced mPFC dendritic complexity and reduced Re volume, we hypothesized that exposure to alcohol in a rodent model of third-trimester binge drinking would shorten the cumulative length of mPFC axon terminals within Re. This study adds further examination of how this integral circuit is impacted by developmental AE.

## Materials and methods

### Experimental subjects

All procedures were carried out in accordance with the University of Delaware Institutional Animal Care and Use Committee (IACUC) and with the NIH’s Animal Care Guidelines. A total of 31 male and female Long Evans rats generated from 7 litters were used (male = 16, female = 15). All animals were born at the University of Delaware. On postnatal day (PD) 3, all animals were paw marked for identification with black India ink subcutaneously injected into the paw pad. A timeline depicting all animal experimental procedures is depicted in Figure 1B.

### Postnatal treatments and blood alcohol concentration analysis

On PD 4, pups were randomly assigned to one of two postnatal treatment groups: alcohol exposed (AE) (*n* = 16; male = 8, female = 8) and sham intubated (SI) procedural controls (*n* = 15; male = 8, female = 7). No more than two pups of either sex were used from each litter for both postnatal treatments. AE pups were administered alcohol in milk formula (5.25 g/kg/day, two doses separated by 2 h), from PD 4–9 *via* intragastric intubation (for milk formula recipe, see West et al., 1984). SI pups underwent all procedures performed on AE pups but were not administered any liquid during intubation. AE pups received a third supplemental dose of milk substitute 2 h after the final dose on each day to promote healthy growth which would otherwise be hindered by intoxicated pups’ inability to suckle from the dam.

Blood samples (~ 60 μL) were collected from all pups *via* tail clip 90 min after the second alcohol dose on the first day (PD4) to assess peak blood alcohol concentration (BAC). Blood samples of SI pups were discarded. Blood from AE pups were immediately centrifuged at 15,000 × g at 4°C for 45 min. Plasma was then collected and BACs were analyzed using an Analox GL5 Analyzer (Analox Instruments, Boston, MA). The mean (± SEM) BAC of AE pups was 250.075 ± 31.68 mg/dL, which is comparable with other studies using similar dosing methods (Thomas et al., 2008; Gursky et al., 2020a; Gursky and Klintsova, 2021; Milbocker and Klintsova, 2021).

After the dosing period during PD 4–9, animals from both groups were left undisturbed with their respective dams until PD 23, when they were weaned and socially housed (2 animals/cage) into cages with partners of the same sex and opposite postnatal treatment condition. All rats were then left undisturbed until the time of viral tracer injection.

### Viral tracer injection

Stereotaxic surgery was utilized to inject viral tracers into layer V of the mPFC (Figure 1B), which took place between PD 42–46. All animals were weighed and anesthetized *via* inhalation of isoflurane before being head-fixed in a stereotaxic frame (Stoelting Company, product # 51950). 1–3% isoflurane anesthesia was maintained throughout the rest of the procedure. Rats were kept on a heating pad through the duration of procedure and recovery. Eye-lubricant was applied, and body temperature was monitored during the procedure. Adequate anesthetic plane was maintained by monitoring breath rate and absence of involuntary reflexes to pain, such as response to toe pinch. Rats were given subcutaneous injections of 50 mg/mL Carprofen (5 mg/kg) and 0.5% Lidocaine (1 mg/kg) immediately before incision.

After sterilization and incision at the surgical site, a burr hole was drilled into the skull at 3.00 mm anterior and -1.50 mm lateral of bregma to target the left mPFC. All injections were made at a 16°angle in order to avoid damage to the sagittal sinus. Because of the configuration of the stereotaxic apparatus preventing to angle from the right, only injections into the left hemisphere of each rat were made.

A total of 400 nL of AAV5-CAG expressing tdTomato was taken up into a 1 μL syringe (Hamilton Company, product # 65458-01) attached to an automated syringe pump (Harvard Apparatus, product # 70-4507). The syringe needle was slowly lowered 3.00 mm into the burr hole at approximately 0.1 mm/sec. The syringe was left in place for 5 min before the virus was infused at a rate of 100 nL/min using, followed by another 5 min still period to ensure proper virus diffusion. The syringe was then removed at a rate of approximately 0.1 mm/s and the surgical site was sutured closed and covered with triple antibiotic ointment.

pAAV-CAG-tdTomato (codon diversified) was obtained from Addgene (Watertown, MA, United States) and was deposited to the Addgene repository by Edward Boyden (Addgene viral prep # 59462-AAV5; RRID:Addgene_59462).^1^

Following surgery, all animals were single-housed and left undisturbed to recover until 10 days post-op (PD 52–56), after which they were socially housed with the same cage mates prior to surgery. No complications were present during any of the surgical procedures. All animals were then left undisturbed in their home cages until tissue collection.

### Tissue collection and fixation

On PD 70 (24–28 days after virus injection) all rats were deeply anesthetized with isoflurane and intraperitoneally administered a veterinarian-approved mixture of ketamine and xylazine. Upon heavy anesthesia and lack of response to toe pinch, rats were perfused transcardially with approximately 150 mL of heparinized 0.1 M phosphate buffered saline (PBS) (pH = 7.20) followed by approximately 250 mL of 4% paraformaldehyde in 0.1M PBS (pH = 7.20). Upon extraction, brains were postfixed in 4% paraformaldehyde solution for 48 h and later transferred to a solution of 30% sucrose in 4% paraformaldehyde three times before being stored at 4°C until cryosectioning. Brains were allowed to sit in each 30% sucrose/paraformaldehyde solution until the brains sunk to the bottom of the container (24–72 h). This has been shown to indicate successful infiltration of the tissue to prevent freezing artifacts during cryosectioning (Rosene et al., 1986).

After postfixing, brains were systematically sectioned coronally at 40 μm on a Leica cryostat at −20°C. Sections were stored with their order preserved in a sucrose/ethylene glycol cryoprotectant solution at −20°C until fluorescent labeling took place.

### Fluorescent labeling and microscopy

Starting in at a random section within the first eight sections containing prefrontal cortex, every eighth serial section was pulled for florescent labeling. Brain tissue was washed in deionized H_2_O (diH_2_O) and 0.1M PBS, followed by a 0.4μg/μL concentration of Hoechst33342 in 0.1 M PBS. The nucleic acid marker Hoechst33342 was applied to brain sections (representative sections displayed in Figures 1C–E) to visualize and locate regions of interest. After fluorophore application, the tissue was washed in 0.1 M PBS two times before being mounted on unsubbed microscope slides and cover slipped with Gelvatol mounting medium. Slides were left to dry at room temperature for approximately 24 h before being stored at 4°C.

Microscopy images were acquired using a Zeiss AxioImager M2 microscope with Apotome and Colibri 7 LED illumination (Carl Zeiss AG, Oberkochen, Germany). 3D image stacks used for cumulative axon terminal length analysis were acquired using a high-numerical aperture (NA) 63 × oil immersion objective (NA = 1.40, Plan Apochromat, product # 420780-9900-000) with Apotome, while images used for estimation of injection volume and Re volume were acquired with a 5 × non-immersion objective (NA = 0.16, Plan-Neofluar, product # 420330-9901-000 without Apotome. Fluorophores were excited using adjustable-intensity light emitting diodes with single bandpass filter cubes for DAPI/Hoechst (Carl Zeiss AG, Oberkochen, Germany, Filter set 96 HE, product # 489096-9100-000) and tdTomato/Alexa Fluor 568 (Carl Zeiss AG, Oberkochen, Germany, Filter set 43 HE, product # 489043-9901-000).

### Unbiased stereology

Unbiased stereology was used to obtain estimations of cumulative length of mPFC axon terminals within Re, volume of virus injection spread, and Re volume. Unbiased stereology is a method that uses random, systematic sampling to provide accurate estimations of volume, length, and total number of structures of interest (e.g., axons, cells) within an object, such as brain cortical area or subcortical nucleus (West, 2012). We were able to examine these features with high reliability as demonstrated by low observed coefficients of error (CEs), which are presented in Table 1.

**TABLE 1:** Mean Gundersen (m = 1) coefficients of error (CEs).

Sex	Postnatal treatment	CE for mPFC injection Diffusion volume measures (Mean CE ± SEM)	CE for mPFC-originating Axon terminal length (Mean CE ± SEM)	CE for Re volume measures (Mean CE ± SEM)
Female	SI	0.018 ± 0.002	0.049 ± 0.005	0.034 ± 0.003
	AE	0.022 ± 0.003	0.064 ± 0.007	0.039 ± 0.005
Male	SI	0.018 ± 0.001	0.053 ± 0.005	0.032 ± 0.002
	AE	0.019 ± 0.003	0.068 ± 0.008	0.038 ± 0.006

Stereological estimates included diffusion of AAV-CAG injections in mPFC, cumulative length of mPFC-originating axon terminals in Re, and Re volume. It is widely accepted to accept CE values as reliable if below 0.100 (Slomianka and West, 2005).

Nucleus reuniens tracings were made following anatomical landmarks in Paxinos and Watson (2013). Throughout the extent of the brain, Re was identified and outlined from approximately 1.20 mm caudal to bregma to approximately 3.36 mm caudal to bregma. In the most rostral sections, the medial border of Re runs against the paraventricular thalamic nucleus with the lateral border following the bed nucleus of the stria terminalus and the dorsal and ventral edges starting at the anteromedial thalamic nucleus and the paraventricular nucleus of the hypothalamus, respectively. Starting at approximately 1.92 mm caudal to bregma, the dorsal border follows the edge of the rhomboid nucleus of the thalamus with the ventral border along the paraxiphoid nucleus of the thalamus. The lateral borders are distinguished by the visible boundary between the submedium thalamic nuclei and ventral reuniens. These landmarks were used until approximately 2.92 mm caudal to bregma, where the ventral border is replaced by prosomere 3.

In this experiment, the StereoInvestigator software was used to perform two different stereology probes: the Cavalieri and the Spaceballs probes (MBF Bioscience, Williston, VT). The Cavalieri probe was used to estimate injection volume and Re volume, utilizing a systematic random sampling grid of points in 100 μm × 100 μm intervals and counting each point that falls within the region of interest. The Spaceballs probe was used to estimate cumulative length of mPFC-originating axon terminals within Re, which places a 3D sphere within the 3D virtual tissue image stacks at systematic random sites and counts the intersections of axonal structures with the surface of the sphere to calculate overall length density (illustrative schematic in Figure 1F). Spaceballs parameters were as follows: grid size: 237 × 237 μm; sphere radius: 15 μm. Due to the projections from mPFC to Re being strictly ipsilateral (Varela et al., 2014; Hallock et al., 2016), as well as the unilateral injections employed in this study, only the left side of Re was examined.

### Statistical analysis

All statistical analyses were performed using GraphPad Prism version 9.3.1. An α = 0.05 significance level was used for all statistical tests. All response data had homoscedasticity of variance. All response data met the assumption of normality as assessed by Shapiro-Wilk tests besides the weight data for male SI rats obtained during time of virus infusion (PD42-46). However, because ANOVA has been shown to be quite robust against non-normality (Blanca et al., 2017), and the lack of a suitable non-parametric test for 2 × 2 factorial design, parametric tests were chosen to analyze weight data at this time point (for normality test results, see Supplementary Table 1). For all analyses, a 2 × 2 ANOVA was performed with sex and postnatal treatment as independent variables. Grubbs’ test for outliers was performed on all data subsets, and two data points were excluded from the axon terminal length analysis after deemed significant outliers. Bonferroni-corrected *post-hoc* tests were employed where indicated.

## Results

### Animal weights

Animal weights at time of virus infusion and at time of perfusion were analyzed to investigate the impact of postnatal treatment on growth rate throughout the course of this study. Two 2 × 2 ANOVAs revealed significant main effects of sex at both the earlier and later timepoint, [*F*(1, 27) = 149.1, *p* < 0.0001, η_*p*_^2^ = 0.847] and [*F*(1, 27) = 161.5, *p* < 0.0001, η_*p*_^2^ = 0.857], respectively, which was to be expected as male rats weigh more than female rats beginning in adolescence and persisting through adulthood. At the time of virus infusion, there was a significant main effect of postnatal treatment on body weight [*F*(1, 27) = 5.161, *p* = 0.0313, η_*p*_^2^ = 0.160]. However, this effect was not found when looking at animal weights in adulthood at time of perfusion [*F*(1, 27) = 0.678, *p* = 0.4175, η_*p*_^2^ = 0.024], indicating that effects of postnatal alcohol exposure on animal growth were transient and did not last beyond adolescence, when the brains were collected. These data are represented in Table 2.

**TABLE 2:** Weights (g) of all animal groups.

Sex	Postnatal treatment	Weight at PD 42–46 (Mean ± SEM)	Weight at PD 70 (Mean ± SEM)
Female	SI	180.286 ± 5.656	262.857 ± 4.378
	AE	171.625 ± 5.268	248.125 ± 9.893
Male	SI	263.375 ± 5.815	396.000 ± 12.620
	AE	243.250 ± 7.995	391.750 ± 12.878

	**Group comparisons**	***P*-value (PD 42–46)**	***P*-value (PD 70)**

	Sex	****	****
	Postnatal treatment	*	ns

*p ≤ 0.050, ****p ≤ 0.0001.

### Volume of virus diffusion

A 2 × 2 ANOVA was performed to investigate the effect of postnatal treatment and sex on virus injection volume. No impact of postnatal treatment or sex was observed on injection volume/spread, thus indicating similar viral distribution between animals. The estimated volume of spread for one AE female was quite small due to mPFC tissue being damaged during cryosectioning. Thus, this data point was excluded for this analysis. However, this animal did not vary on other measures such as cumulative axon length or Re volume.

### Reuniens volume

**FIGURE 2:**
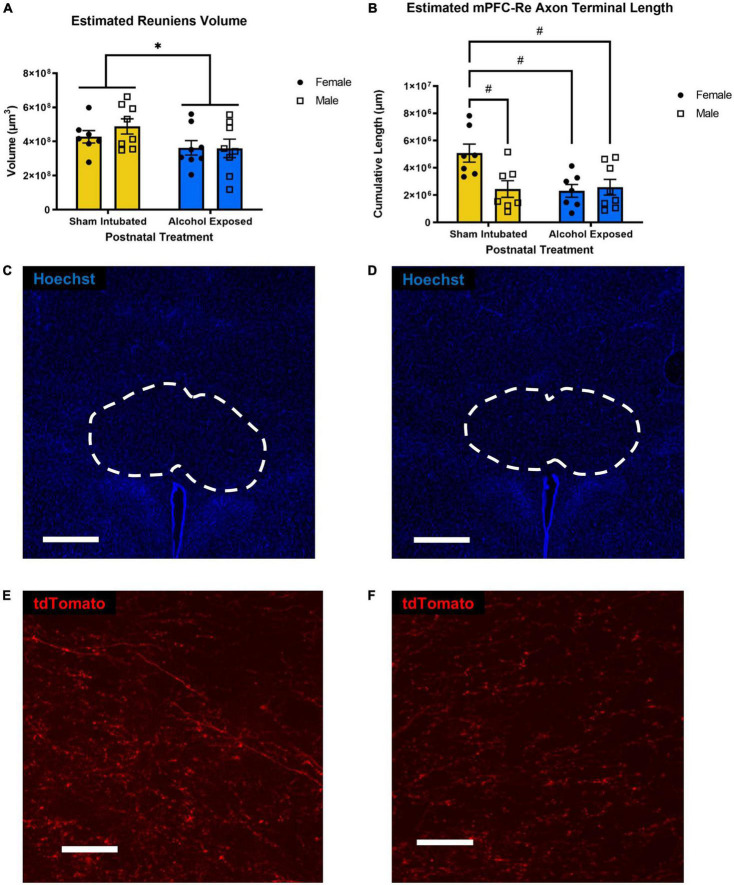
Estimated left Re volume **(A)** and estimated length of mPFC-originating axon terminals within Re **(B)**. Individual data points represent single animals. High-dose alcohol exposure on PD4-9 resulted in reduced total volume of Re in both sexes and reduced length of mPFC-originating axon terminals in female, but not male animals. Data are presented as means ± SEM. *p ≤ 0.05 significant main effects; ^#^*p* ≤ 0.05 significant *post hoc* corrected tests. Representative sections containing Re from a sham-intubated **(C)** and alcohol-exposed animal **(D)** (whole Re outlined) (scale bars = 500 μm), representative sections of magnified Re containing AAV-expressing axonal material from a sham-intubated **(E)** and alcohol-exposed animal **(F)** (scale bars = 1,000 μm).

A 2 × 2 ANOVA was performed to investigate the effect of postnatal treatment and sex on Re volume. A main effect of postnatal treatment [*F*(1, 27) = 4.759, *p* = 0.038, η_*p*_^2^ = 0.143] was found. There was no significant main effect of sex on Re volume, as well as no significant interaction between postnatal treatment and sex. Upon investigation of group means, AE reduced Re volume, which is similar to previous findings from our lab (Gursky et al., 2019; Gursky and Klintsova, 2021, 2022). These data are represented in Figure 2A.

### Length of medial prefrontal cortex-originating axon terminals within reuniens

A 2 × 2 ANOVA was performed to investigate the effect of postnatal treatment and sex on cumulative length of axon terminals projected from mPFC located in Re. No significant main effects of sex [*F*(1, 25) = 4.142, *p* = 0.053, η_*p*_^2^ = 0.142] or postnatal treatment [*F*(1, 25) = 5.082, *p* = 0.033, η_*p*_^2^ = 0.169] were found. However, there was a statistically significant interaction between the effects of postnatal treatment and sex [*F*(1, 25) = 6.189, *p* = 0.0199, η_*p*_^2^ = 0.198]. Bonferroni-corrected *post hoc* analyses revealed that SI Females had significantly higher cumulative axon terminal length than SI Males (*p* = 0.0253), AE Females (*p* = 0.0174), and AE Males (*p* = 0.0296). These data are represented in Figure 2B. Representative sections illustrating differences in both Re volume and labeled axons’ distribution are displayed in Figures 2C–F.

## Discussion

The purpose of the current study was to examine the impact of early postnatal alcohol exposure in an animal model of third trimester binge drinking on mPFC-Re circuitry and Re volume. Our results demonstrate that female AE rats had significantly reduced cumulative mPFC-originating axon terminal length within the nucleus reuniens than female SI rats. However, mPFC-originating axon terminal length in Re of male SI rats did not differ significantly from male or female AE rats. In accordance with previous work from our lab, AE rats exhibited lower Re volumes, regardless of sex.

Results of our study demonstrate a significant effect of alcohol exposure, i.e., the reduced total length of axonal projections within the Re, in female rat brains only. In the brains of both SI and AE male rats, the total length of mPFC axons in Re was significantly reduced compared to total axon length in female SI brains, making them similar to the ones in the Re of AE female rats. These findings suggest that the effect of stress due to postnatal intubation to deliver alcohol could have been stronger in developing male brains than the effect of alcohol alone.

Previous studies on the effects of AE on Re volume and its projections to mPFC and HPC revealed no difference between SI and suckle control animals, and thus the latter group was not included in the present study (Gursky and Klintsova, 2021). However, human literature exists demonstrating that females may be more resilient to prenatal and early life stress (Hodes and Epperson, 2019). Work in rodents have also demonstrated differential sex-driven effects of prenatal stress on brain development (for review, see Weinstock, 2007). Indeed, prenatally stressed male rat pups exhibit significant dendritic atrophy in prefrontal areas, while stressed females do not express such dendritic atrophy (Murmu et al., 2006). Additionally, a rodent model of early life stress taking place during the third trimester equivalent of brain development results in no reduction in neurogenesis in female rats, suggesting that female brains are more resilient to stress insults at this time compared to males (Loi et al., 2017). These studies support the idea that the observed decrease in cumulative axon length in SI males may be due to male rat pups being more sensitive to intubation stress, which is a familiar concern in FASDs literature (Kelly and Lawrence, 2008).

Because measurements from suckle control animals are lacking, it is also possible that a sex difference at baseline could explain the observed dissimilarity in mPFC axon length between SI males and SI females. It has been shown that female rats exhibit greater arborization of dendritic branches in agranular insular regions of the PFC than males, while the reverse was found in cingulate areas (Kolb and Stewart, 1991). Additionally, sex differences in the amount of myelinated axonal material have been reported. Adult female rats were found to have higher levels of both myelin basic protein and myelin proteolipid protein in the orbitofrontal cortex than adult males (Bayless and Daniel, 2015). Bayless and Daniel also showed that females had a greater number of efferent projections from the orbitofrontal cortex to the dorsal striatum than males. While this is a different subregion of the rodent prefrontal cortex, these experiments suggest a baseline sex difference in that females have both more myelinated axons and direct projections in the prefrontal cortex than males. Taken together, this could mean that the SI males in the current study may exhibit shorter mPFC-originating axon terminals in Re than females in the absence of intubation stress. Although a baseline sex difference in axonal material could be present, it is interesting that a reduction in Re volume was found in both sexes as a result of AE. The differential effects on brain regions vs. circuitry should be taken into consideration in the future when assessing the impact of alcohol exposure.

These results may also be explained by sex-specific characteristics of microglia. It has been established that male rodents have more microglia during third trimester equivalent brain growth than females (Schwarz et al., 2012). Additionally, adult male, but not female, mice that experienced an early immune challenge exhibit increased cellular stress and microglia-synapse interactions, which may give a reason for the severe effect of intubation stress in males observed in the current study (Hui et al., 2018).

One possible explanation to the resulting pathology of FASD is that many neurodevelopmental disorders are linked to an imbalance of excitation and inhibition during early life (Fernandez and Garner, 2007; Coghlan et al., 2012). Previous work from our lab has established that AE brings on immediate death of neurons in Re that lasts long into adulthood (Gursky et al., 2020b). However, we have also demonstrated that AE leads to an increase in proportion of Re neurons that collaterally project to both mPFC and HPC without altering the number of neurons that project to only one of these two structures (Gursky and Klintsova, 2021). Thus, AE may be creating an imbalance of excitatory neurons in the Re that could ultimately affect the anatomical substrates of the mPFC-Re-HPC circuit (i.e., mPFC axonal projections). Work from our lab has already shown that AE leads to altered dendritic complexity and spine density in mPFC (Hamilton et al., 2010). For now, however, it remains unclear whether changes to mPFC fibers result from the direct effect of AE, or if they are secondary to changes to their connecting structures.

While we have demonstrated alcohol-induced alterations to mPFC-Re axonal projections many other subcortical regions receive projections from layers V/VI of the mPFC (Vertes, 2002). Although Re is more specifically affected by AE than neighboring thalamic nuclei (Gursky et al., 2019), measuring axon terminal length in other mPFC targets could discern the specificity of the effect of alcohol exposure on different mPFC efferent pathways.

Now that mPFC-Re projections’ alterations have been established, future studies should utilize anterograde tracers in both mPFC and HPC to map innervation (and potential co-innervation) of Re neurons from these two structures. It is also important to investigate behavioral interventions that might potentially mitigate these anatomical deficits. There is an urgent need for effective therapeutic interventions for FASD. Despite this clear need, there exist only a small number of clinical randomized studies that investigate the efficacy of interventions created to improve the cognitive and behavioral outcomes of individuals diagnosed with FASD. It is important to design targeted interventions aimed at specific behavioral and cognitive facets, such as EF. There have already been studies reporting that exercise can enhance EF in human children (Chang et al., 2012; Lambrick et al., 2016). Animal models can provide critical investigation as to how interventions that supposedly target EF affect the neuronal substrates underlying executive function, such as mPFC-Re-HPC circuitry.

A limitation to this study was the inability of the anterograde viral tracer to cross synapses. Because of this, it was not possible to label and quantify the population of neurons in Re that receive input from mPFC. It was also impossible to determine the proportion of AAV5-CAG-expressing neurons in the mPFC that project to Re vs. other efferent targets. Furthermore, we are limited in our interpretation of the cumulative axon terminal length results due to the typical features and nature of the Spaceballs probe. Because this stereological probe provides a measurement of overall length of fibers, we currently can only determine that the cumulative length of axonal material is affected rather than other aspects of the axons (e.g., number, complexity, etc.).

Future directions of this study include correlating these results with deficits in EF. Behavioral tasks that are known to rely on mPFC-Re-HPC circuitry, such as the object-in-place task (Barker and Warburton, 2018), can be utilized to provide a functional outcome of observed neuroanatomical results. Additionally, it is currently unclear how developmental alcohol exposure affects the functional connectivity of the mPFC-Re-HPC circuitry, which can be assessed in the future by utilizing electrophysiological recording coupled with circuit-dependent behavioral tasks. While these experiments have yet to be completed, this study was an additional necessary step in helping us better understand how perinatal AE affects mPFC-Re-HPC connectivity and subsequent EF deficits associated with FASDs.

## Data availability statement

The raw data supporting the conclusions of this article will be made available by the authors, without undue reservation.

## Ethics statement

The animal study was reviewed and approved by the University of Delaware Institutional Animal Care and Use Committee.

## Author contributions

AK and ZG: conceptualization and funding acquisition. ES and ZG: methodology. ES: formal analysis, writing—original draft preparation, and visualization. ES, AK, and ZG: writing—review and editing. All authors contributed to the article and approved the submitted version.

## Footnotes

1
http://n2t.net/addgene:59462
