# Supplementary material for: Representation of prefrontal axonal efferents in the thalamic nucleus reuniens in a rodent model of fetal alcohol exposure during third trimester
Source: Front Behav Neurosci. 2022 Sep 8;16:993601. doi: 10.3389/fnbeh.2022.993601 (PMC9493097; doi:10.3389/fnbeh.2022.993601)
Supplement: Supplementary file 1 [file Table_1.DOCX]

Supplementary Table 1. Shapiro-Wilk normality test results for all statistical groups for all analyses.

| **mPFC-Originating Axon Terminal Length** | | | | |
| --- | --- | --- | --- | --- |
| **Sex** | **Postnatal Treatment** | **Statistic (Shapiro-Wilk)** | **Degrees of Freedom** | **Significance** |
| Female | SI | 0.873 | 7 | 0.197 |
|  | AE | 0.966 | 7 | 0.867 |
| Male | SI | 0.880 | 7 | 0.226 |
|  | AE | 0.849 | 8 | 0.093 |
|  |  |  |  |  |
| **Reuniens Volume** | | | | |
| **Sex** | **Postnatal Treatment** | **Statistic (Shapiro-Wilk)** | **Degrees of Freedom** | **Significance** |
| Female | SI | 0.944 | 7 | 0.678 |
|  | AE | 0.899 | 8 | 0.282 |
| Male | SI | 0.882 | 8 | 0.196 |
|  | AE | 0.953 | 8 | 0.746 |
|  |  |  |  |  |
| **Injection Diffusion Volume** | | | | |
| **Sex** | **Postnatal Treatment** | **Statistic (Shapiro-Wilk)** | **Degrees of Freedom** | **Significance** |
| Female | SI | 0.957 | 7 | 0.792 |
|  | AE | 0.949 | 7 | 0.722 |
| Male | SI | 0.956 | 8 | 0.775 |
|  | AE | 0.902 | 8 | 0.301 |
|  |  |  |  |  |
| **Animal Weight at PD 42-46** | | | | |
| **Sex** | **Postnatal Treatment** | **Statistic (Shapiro-Wilk)** | **Degrees of Freedom** | **Significance** |
| Female | SI | 0.950 | 7 | 0.728 |
|  | AE | 0.989 | 8 | 0.994 |
| Male | SI | 0.771 | 8 | 0.014 |
|  | AE | 0.972 | 8 | 0.912 |
|  |  |  |  |  |
| **Animal Weight at PD 70** | | | | |
| **Sex** | **Postnatal Treatment** | **Statistic (Shapiro-Wilk)** | **Degrees of Freedom** | **Significance** |
| Female | SI | 0.869 | 7 | 0.180 |
|  | AE | 0.881 | 8 | 0.192 |
| Male | SI | 0.894 | 8 | 0.255 |
|  | AE | 0.892 | 8 | 0.246 |
